# Andrew H. Wyllie, a pioneer in the field of apoptosis

**DOI:** 10.1186/s12964-022-00988-z

**Published:** 2022-10-17

**Authors:** Zahra Zakeri, Richard A. Lockshin

**Affiliations:** 1 Department of Biology, Queens College of CUNY, 65-30 Kissena Blvd., Flushing, NY 11367 USA; 2 Department of Biological Sciences, St. John’s University, 8000 Utopia Parkway, Jamaica, NY 11439 USA

**Keywords:** Apoptosis, DNA ladder, Andrew Wyllie

## Abstract

We mourn, and briefly describe the life and contributions of, Andrew H. Wyllie, who was a co-author of the first paper to describe apoptosis, and a primary proponent of the concept.

With sorrow we announce the passing of Andrew H Wyllie (Fig. 1) on May 26, at age 78. Wyllie was a giant in the field of cell death, having, in a famous paper in 1972 announced, with John Kerr and Alastair Currie, the existence of apoptosis as an unique, widespread, and important biological phenomenon [1] Today apoptosis is considered a fundamental process in early development [2], at many points and especially the central nervous system. It is also a primary aspect of homeostasis. Many cancers derive from failure of the regulation of apoptosis. Aggressive treatment of many diseases includes either trying to prevent apoptosis, as in aging of the immune system and brain, or protecting against the worst ravages of viruses such as Covid-19; or in trying to reactivate apoptotic pathways in many types of cancer.

**Fig. 1:**
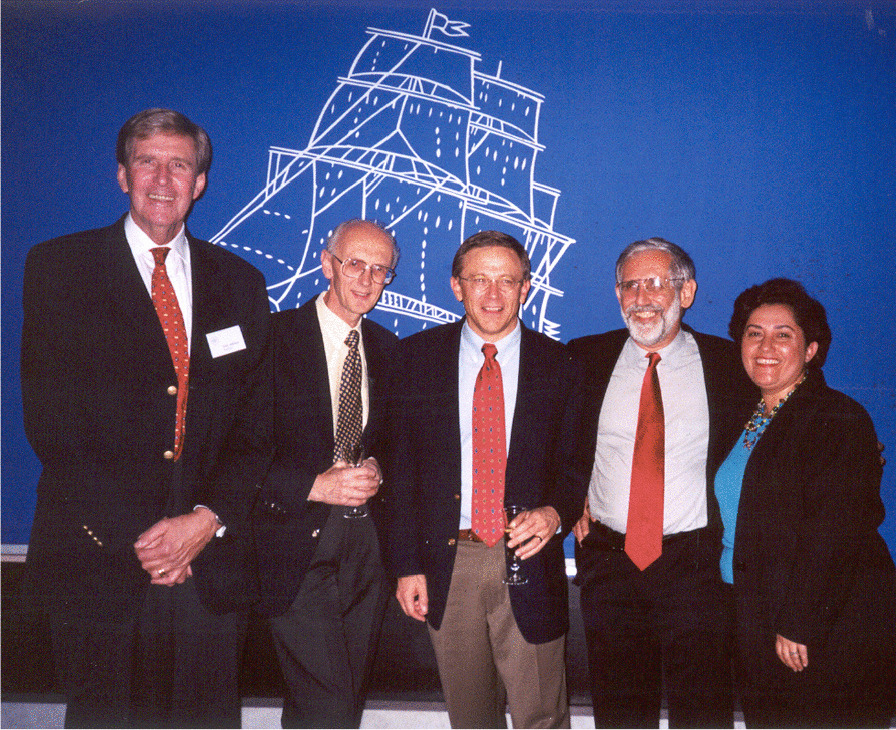
Andrew H. Wyllie at a Nobel Conference in 2001. Left to right: Sten Orrenius, Andrew Wyllie, Stan Korsmeyer, Richard Lockshin, Zahra Zakeri

Wyllie later emphasized the characteristics and the importance of apoptosis [3]. In a third and fourth important papers, he emphasized the relationship of the ladder of fragmented DNA that characterized apoptosis, thus providing an inexpensiveand easily reproducible means of assessing apoptosis and allowing other researchers to look for the phenomenon [4, 5] (Fig. 2). At the time of his death, he had been cited over 70,000 times, and the term apoptosis figured in over 900,000 publications. He was a member of many honorary societies. He died at home, surrounded by his family. His memory will be cherished.

**Fig. 2:**
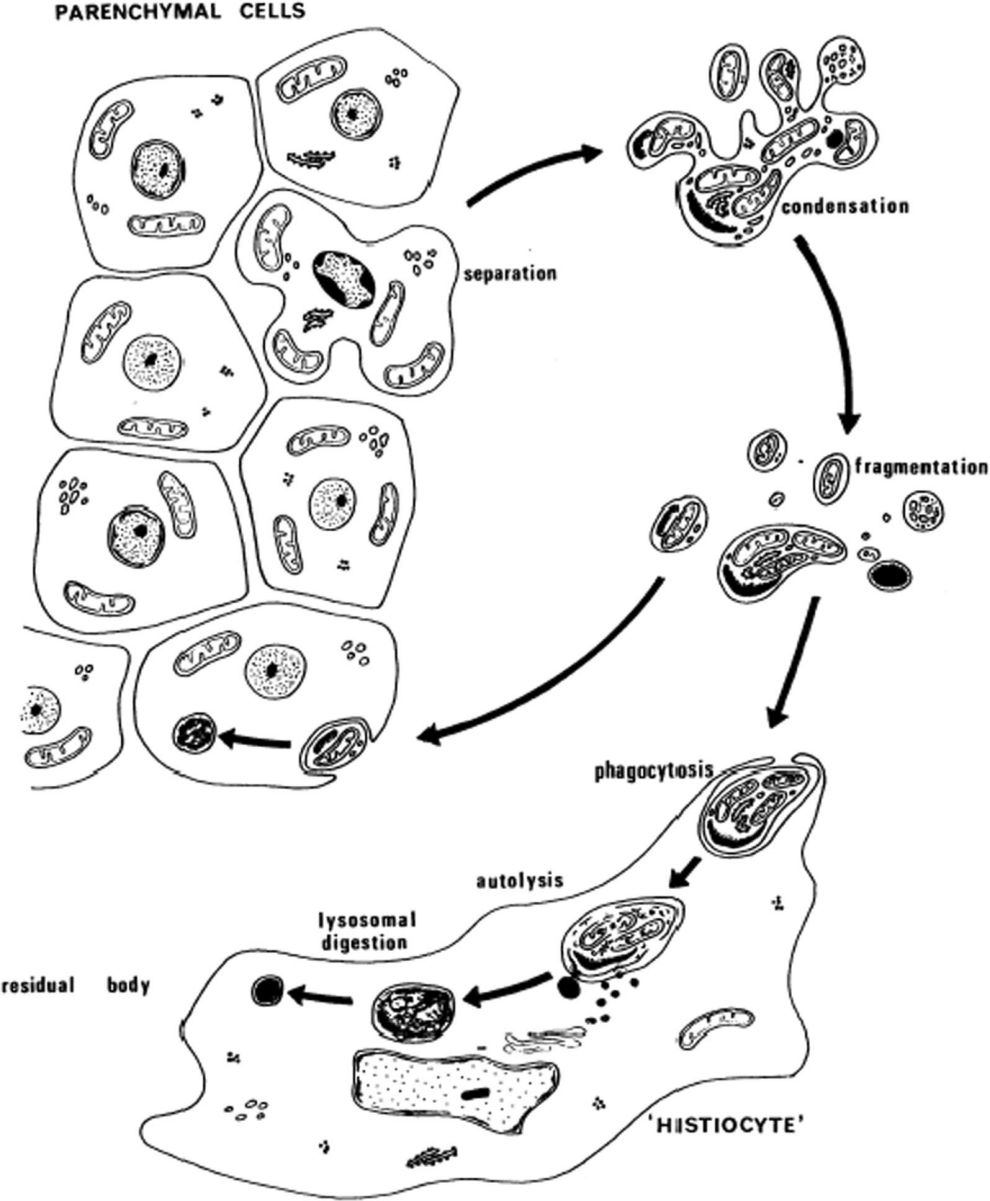
Original scheme of apoptosis as described by Kerr, Wyllie, and Currie [1972]. As defined by Wyllie and others, typical apoptosis is now considered to include Cleavage of HMW DNA, Chromatin condensation, Chromatin margination to nuclear membrane, Nuclear membrane shrinkage, Oligonucleosomal cleavage of DNA, Fragmentation of nucleus, Cytoplasmic shrinkage, Rounding of cells, Formation of vacuoles in the cell, Exteriorization of phophatidylserine, Activation of caspase-3

## Data Availability

Figure 2 is available online at PubMed. Figure 1 is the property of the authors and is available on request.
